# Connection of ES Cell-derived Collecting Ducts and Ureter-like Structures to Host Kidneys in Culture

**DOI:** 10.1080/15476278.2021.1936785

**Published:** 2021-09-27

**Authors:** May Sallam, Jamie Davies

**Affiliations:** a Deanery of Biomedical Science, University of Edinburgh, Edinburgh, UK; b Human Anatomy& Embryology Department, Faculty of Medicine, Mansoura University, Mansoura, Egypt

**Keywords:** Regeneration, renal, ureter, organoid, tissue-engineering, anastomosis

## Abstract

Work toward renal generation generally aims either to introduce suspensions of stem cells into kidneys in the hope that they will rebuild damaged tissue, or to construct complete new kidneys from stem cells with the aim of transplanting the engineered organs. In principle, there might be a third approach; to engineer renal tissue ‘modules’ in vitro and to use them to replace sections of damaged host kidney. This approach would require the urine collecting system or ureter of the new tissues to connect to those of the host. In this report, we demonstrate a method that allows collecting duct trees or ureters, engineered from ES cells, to connect to the collecting duct system or ureter, respectively, of fetal kidneys in culture.

## Introduction

Chronic renal disease is a relatively common and serious condition.^1^ When it proceeds to end-stage renal disease, there are only two treatment options: frequent renal dialysis, or kidney transplantation. The latter is associated with a much higher quality of life than the former, but its application is limited by the availability of suitable donor kidneys and by the need to prevent rejection, through a combination of tissue matching and immunosuppressive drugs.^2^ There is therefore significant and growing interest in growing replacement kidneys from a patient’s own stem cells, with or without correction of any underlying genetic defects.^3^

Progress so far has achieved the production of simple renal organoids that begin with pluripotent stem cells and recapitulate kidney development by guiding stem cell differentiation using sequences of growth factors and drugs.^4–7^ The resulting organoids provide useful models for studying development and disease, and for drug screening. Furthermore, if they are implanted in vivo the organoids even become vascularized,^8,9^ but they are very small (of the order of 1 mm) and they cannot be grown larger in culture by any current technology.

One intriguing alternative to trying to grow organoids large enough to replace a complete kidney would be to use multiple organoids (or other kinds of engineered renal tissue) as modules to replace pieces of a diseased kidney, by grafting them into it. This would only work if it were possible for the organoid to ‘plumb itself in’ to the urinary collecting system so that urine made by it (thanks to blood supply we already know can be created by the host)^8,9^ has somewhere to drain. This paper demonstrates that ureteric buds (collecting duct/ureter progenitors) engineered from pluripotent cells and differentiated into collecting ducts can connect with the urine collecting duct system of host kidneys in culture, and those differentiated into ureters can connect to the ureter of host kidneys in culture.

The work has its roots in our previous study,^10^ in which we developed a method for generating ureter tissues from pluripotent stem cells that had been differentiated into ureteric buds. In this study, we showed that, if the stem cell-derived ureteric buds were grafted into the cortex of a host fetal kidney in vitro, they would differentiate into collecting ducts, but if they were grafted into peri-Wolffian mesenchyme, they would make urothelium that cooperated with the host mesenchyme to make spontaneously contractile ureter tissue.^10^ While we were optimizing the grafting techniques necessary for that work, we sometimes inadvertently damaged the host epithelia and, in these damaged samples, we thought we saw occasional cultures in which the graft and host epithelia seemed to connect.

We therefore decided to extend our initially informal observations of apparent connections made between grafts and damaged hosts, to ask whether deliberately damaging host tissue in a methodical manner would encourage connection, and to ask whether the connections are real, with a contiguous lumen, or merely adhesions that make no meaningful connections in terms of fluid flow. We find that fetal kidney or ureter hosts can be induced to accept connections from grafted natural ureteric buds (UBs) or ES-derived engineered ureteric buds (eUBs), by a ‘nicking’ procedure that damages the wall of the host tubule near the graft. The grafted ureteric buds or eUBs integrate and differentiate into structures similar to those to which they connect, and share a continuous open lumen that can be seen using immunofluorescence. In the case of grafts to the ureter, they show contractions synchronous with the host.

This observation, though made using only immature host organs in culture, may be a foundation for developing methods for connecting organoids into mature host kidneys to augment their function.

## Results

### Differentiation of Hoxb7-GFP ES cell-derived eUBs

The eUBs for the experiments we describe here were therefore made by exactly the same procedure as in our report of urothelial differentiation,^10^ the ‘Taguchi protocol’, first described by the Nishinakamura laboratory at Kumamato University, Japan.^11^ Briefly, the Hoxb7-GFP mouse ESC line was maintained on ES maintenance medium^11^ for several days and then differentiated into GFP-eUBs using the sequence of medium changes in the Taguchi protocol.^11^ At day 10, the ES cells formed UB-like tubules that expressed the UB marker hoxb7-GFP (Figure S1 A, B). These structures showed the ability to branch in 3D gel supplemented with branch inducing factors (Figure S1 C, D), as expected from published work.^10,11^

### eUBs can be induced to connect to host collecting ducts

Our previous study, on how to control eUB differentiation into either collecting duct-type or ureter-type epithelia, included experiments in which single eUB epithelial tubes were grafted into various locations of E11.5 host kidney rudiments in culture. The point made by the experiments was that grafting into mesenchyme of the kidney provoked differentiation into collecting duct-type epithelia, while grafting into the peri-Wolffian mesenchyme, through which the natural ureter passes, provoked differentiation into ureter-type epithelia.^10^ In most experiments, the graft remained completely separate from host collecting duct or ureter epithelia. A small number of host kidneys, however, were badly damaged by the manual manipulations involved in grafting, and some of these seemed to show apparent connections between graft eUB-derived and host UB-derived tubules.

**Figure 1.**
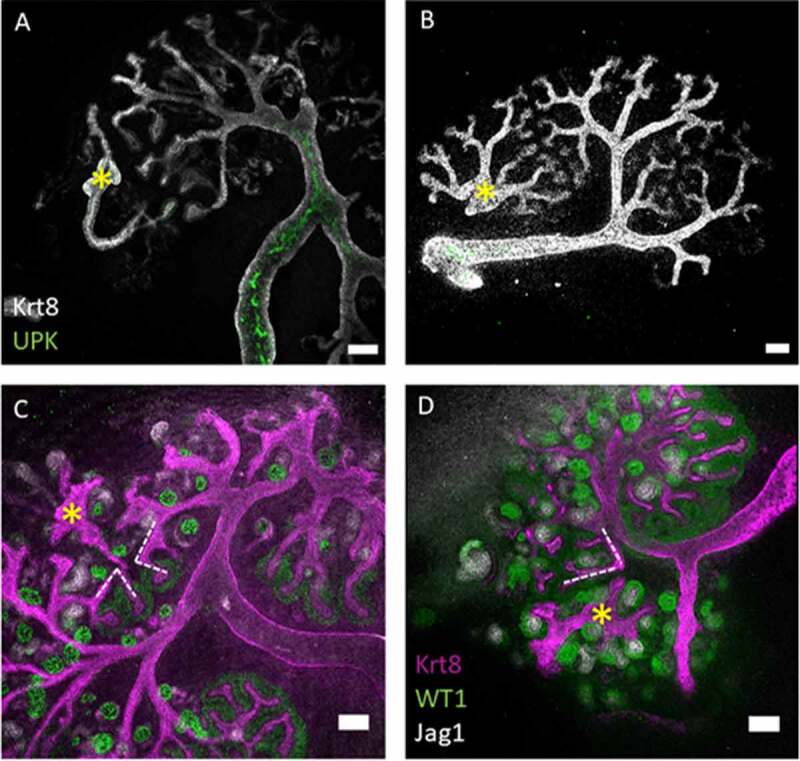
The collecting duct branches of the host kidney avoid contact with the eUB branches. (a–d) Show eUBs (marked with yellow asterisk) grafted in the MM of host kidneys where the CD of the host, facing the graft, stop expanding in A, B or tilt their branches (dotted lines in c, d) to avoid contact with the eUB graft branches. Scale bar = 100 µm.

To test the hypothesis that damage can be used to induce connection between graft and host, we have now set up similar cultures but with deliberate damage to the host epithelium. As in our previous publication,^10^ we made eUBs from hoxb7-GFP mouse ES cells so that the GFP signal could be used to distinguish graft from host. Single tubules of eUBs were dissected from day 10 spheroids (as in Figure S1 A, B) and were grafted into the metanephric mesenchyme of E11.5 host kidneys and cultured for several days on Transwell inserts in standard kidney culture media. In the absence of injury, the grafted eUBs branched and induced nephrons as natural developing collecting ducts do (Figure 1a–d). They remained independent of the host UB-derived epithelium. Moreover, the branches of the collecting duct tree of the host kidney seemed actively to avoid making contact with the graft, growing branches ceasing their advance (Figure1a,b) or apparently curving away to produce locally atypical tree morphology (Figure 1c,d). This was not surprising as repulsion between collecting ducts has been described before.^12^ No connections were seen between graft and host ureteric buds/collecting duct systems (6 samples examined; 0%, CI^95%^ ± 8.3%).

**Figure 2.**
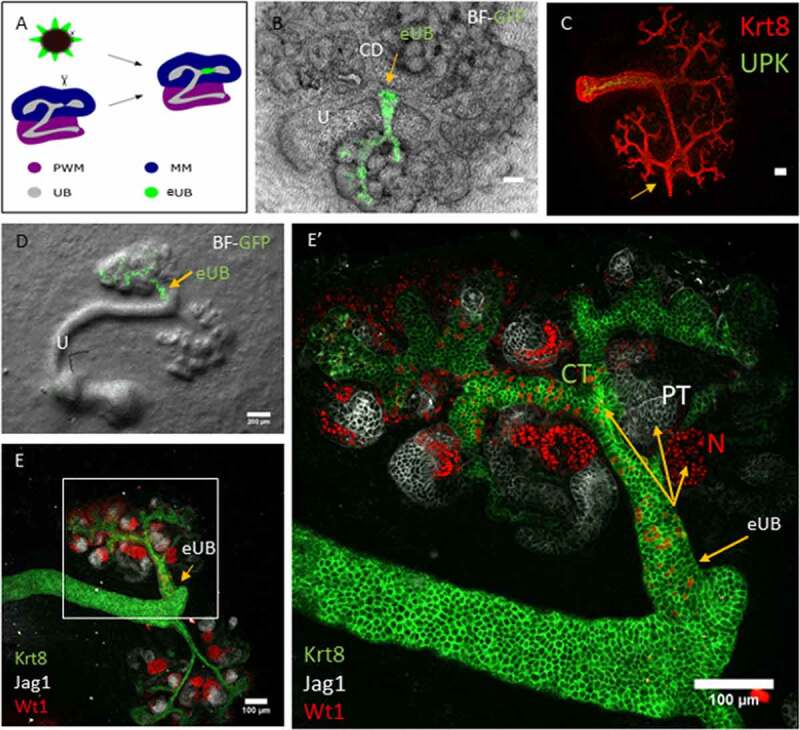
The eUB can be induced to connect to the collecting duct system of a natural kidney. (a) Steps of the process of eUB connection to host kidney CD. (b) Bright field image showing the GFP-eUB connected to the collecting duct tree and showing branching. (c) Immunofluorescence stain of the eUB (arrow), connected to the collecting duct tree and show no UPK expression. (d) Bright field image showing GFP-eUB connected to the collecting duct tree (arrow) and showing branching. (e) Immunofluorescence stain of D, showing eUB branched and induced nephron formation. (E’) Magnified image of E showing the connected eUB surrounded by WT1+ and Jag1+ nephrons. (CD; collecting duct, N; Nephron, PT; proximal tubule).

When a sharp needle was used to make a cut in the host collecting duct (as depicted in Figure 2a), close to the intended graft site and immediately before grafting the eUB next to the cut, connections formed about half the time (Figure 2b,d). In 20 samples examined, 10 showed connections (50%; CI^95%^ ±24%, a range that does not overlap with the 95% confidence interval of the uncut controls described in the paragraph above). As would be expected of collecting ducts, there was no expression of UPK (Figure 2c), but the graft did become surrounded by nephrons that connected to it (Figure 2e).

A time-lapse video recording of the connection process between eUB graft and host collecting duct tree can be viewed in video 1S (contact between the graft and the host could be detected approximately 24 h after grafting and became more evident after 3 days of culture).

### eUBs can be induced to connect to host ureter stalk

**Figure 3.**
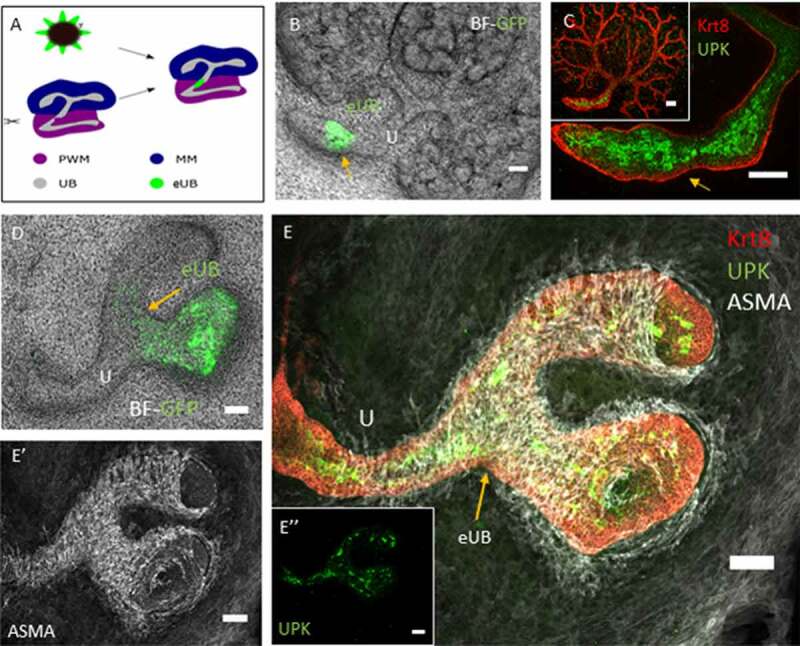
The eUB can be induced to connect to the ureter of a natural kidney. (a) Steps of the process of eUB connection to host kidney ureter. (b) Bright field image showing the GFP-eUB grafted and connected to the ureter (arrow) of a cultured kidney. (c) High-power immunofluorescence image of an eUB connected to the ureter, showing UPK expression, (arrow points to the graft, as in B). (d) Another grafted eUB connected to a host ureter. (e) Immunofluorescence stain of D showing Krt8, UPK and ASMA expression in the graft as well as the host. (E’) Isolated ASMA channel of E, showing smooth muscle coat around the connected graft as well as the ureter (E”) Individual UPK channel shows UPK expression in both the ureter and the connected graft. Scale bar = 100 µm.

Having shown that deliberate cutting of the host collecting duct could promote connection between that host and a nearby grafted eUB differentiating into collecting duct, we asked whether the same was true of host ureter and a grafted eUB developing into urothelium. Again, the host ureter stalk was deliberately nicked with a sharp needle just before the eUB was grafted nearby (Figure 3a). In controls, with no deliberate cut, no connections were seen (6 samples examined; 0%, CI^95%^ ± 8.3%). When a cut was made in the host ureter stalk and the eUB was grafted nearby, connections were seen (20 samples examined, 13 showing connections; 65%, 95%CI± 22.5: this does not overlap the 95% confidence interval of controls). In these connected grafts, the hoxb7-GFP eUB remained unbranched (Figure 3b,d), expressed UPK (Figure 3c,e) and acquired a coat of smooth muscle cells (Figure 3e,E) exactly as occurs in unconnected grafts.^10^

A time-lapse video recording of the connection process between eUB graft and host ureter can be viewed in video 2S (recording started immediately after grafting and lasts for 3 days).

### eUBs connected to a host kidney ureter show synchronous contractions

**Figure 4.**
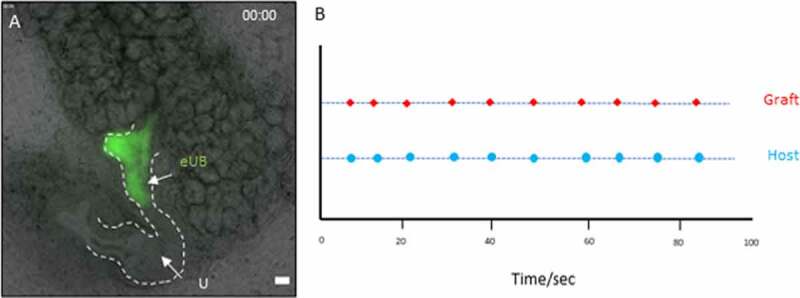
Contraction of the grafted eUB-derived ureter-like tissue and the natural ureter connected to it. (a) shows a starting frame of the video recording (video 3S); the graft which is connected to the host ureter can be identified by the GFP fluorescence, and the arrows indicate the places at which contractions in the graft and the nearby natural ureter were timed. (b) A graph shows the timings at which contractions occurred, shown as dots on the same time-scale.

As we noted in our earlier study,^10^ an eUB grafted in the peri-Wolffian mesenchyme shows spontaneous contractions, of a broadly similar periodicity to those of the host ureter but independently of it in respect to phase and precise frequency. As this suggests an endogenous pacemaker activity in the graft, we wondered what would happen in cultures in which we had used cutting to encourage connection between graft and host. This was investigated using time lapse video recording after 7 days in culture. When eUBs were induced to connect to a host kidney ureter, they showed synchronous contractions with each other, contraction in the graft coinciding with that in the part of the UB to which it had joined (Figure 4, Video 3S). This behavior was seen in 3/3 samples recorded using time-lapse.

### The connected eUB grafts share open lumen with the host collecting duct system

**Figure 5.**
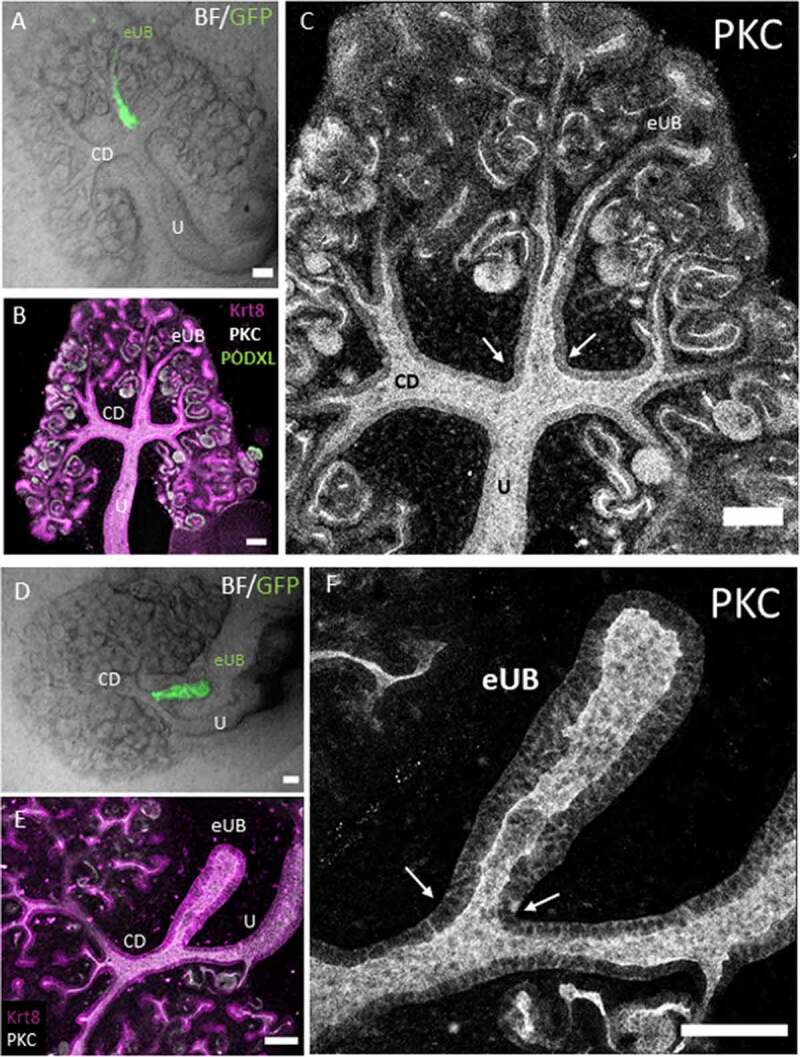
Connections between grafted eUBs and host CD/Ureter show open lumens. (a) Bright field image showing the GFP-eUB connected to the collecting duct system of a host kidney and showing branching. (b) The apical domain protein kinase C (PKC), and the epithelial marker Krt8 stain of the grafted kidney showing a connected lumen between the eUB graft and the collecting duct branches of the host kidney (arrow), early nephrons (expressing PODXL) can be seen connected to the graft. (c) Isolated channel of PKC, for clarity. (d) Bright field image of an GFP-eUB connected to the ureter of a cultured kidney, to show the position of the graft (e) The apical domain PKC and the epithelial marker Krt8 stain showing that the lumen is continuous between the graft and host tubules (arrows). (f) Shows the PKC channel only for clarity. (U: ureter, CD: collecting duct, eUB: engineered UB). Scale bar = 100 µm.

To verify that these eUBs connected to, rather than just attached to, the host tubular system, the boundaries of the lumen were visualized by staining for the apical domain of the epithelia using antibodies to the apical protein, protein kinase C zeta. Confocal images showed the collecting duct and eUB tubule walls joining together to make a continuous wall and leaving a clear luminal space (Figure 5a–c). This was clear in 6/6 examples examined (100%, CI^95%^ ± 8.33%). Similarly, the lumen was continuous across the boundary between eUB grafts and host ureter stalks (Figure 5d–f) in 5/5 examples examined (100%, CI^95%^ ± 10%).

## Discussion

In this paper we describe an unexpected ability of ES cell-derived eUBs to connect to the epithelial tubules of a host kidney, forming a continuous lumen between the graft and the host. The connection was made to collecting ducts or ureter, depending on the site of engraftment, the site also determining the collecting duct/ureter fate of the graft as is already known.^10^

Developing kidneys are rich in signals, likely to be important in determining the differentiation of grafted eUBs.^10^ The metanephric mesenchyme expresses GDNF, which activates the expression of the receptor tyrosine kinase RET and the co-receptor GFRα1 on the UB tip cells to stimulate UB branching, while the UB produces Wnt9b which promotes nephron formation via mesenchymal to epithelial transition.^13^ The future ureter is surrounded by a Tbx18-expressing cell population, the peri-wolffian mesenchyme (PWM), and the UB and the PWM interact to form the urothelium and its contractile machinery.^14^ PWM cells express BMP4, which promotes differentiation of the nearby ureteric bud into urothelium, as well as their own differentiation into smooth muscle cells. The urothelium expresses SHH which binds PTCH1 receptor in the PWM cells to stimulate their proliferation. Both SHH and BMP4 are required for a successful differentiation of the smooth muscle cells.^15^ The possible roles of each of these molecules in specifying eUB differentiation could be tested by a combination of inhibition or ectopic expression/application.

The connection of one ureteric bud derivative to another is not a feature of natural UB/collecting duct ureter development. The entire ureter and collecting duct system develop by branching morphogenesis from the UB, with no need for any connections to be made and with no evidence of connections ever being made. Indeed, the branches of the growing tree show mutual repulsion.^12^ Within the kidney, however, nephrons are required to make a connection between their distal pole and the collecting duct branch that induced their formation in the first place.^16^ It may be that the need to perform this connection, the mechanisms of which are still not understood in detail, means that UB-derived epithelia retain an innate ability to make connections that generate open-lumen communications between the tubules involved.

Damage (nicking) of the host ureter or collecting duct is necessary for connections to form. It should be noted that one end of each eUB will also have been cut, as an inevitable step in its isolation from its parent branched system. There was no feasible way of tracking, during grafting, which end of the UB was the cut one, so whether the intact or the cut end of the eUB was opposed to the nicked host epithelium was random. The approximately 50% success rate of making connections in all of these experiments would be compatible with the idea that connection requires both of the apposed tissues to have been cut before apposition. There are several possible reasons that damage may be essential. One is mechanical; if two undamaged tubes met, they would do so basal surface-to-basal surface, and there would be no obvious thermodynamic (reduction of free energy through better adhesion) or other reason for their cells to exchange existing neighbor relationships for new neighbor relationships with cells of the other epithelium. With a gap in at least one side of the host tubule, cells adjacent to the gap will go on to make a new adhesion with something, either to cells of their own kind across the gap, or to a cell of the graft, and this might raise the probability of host-graft connections being made instead of host-host healing. There may also be reasons associated with the production of signaling molecules.

Normally, close apposition of UB-derived epithelia is prevented by their secreting and avoiding BMP7,^12^ for example, when two UB epithelia were cultured very close to each other, they try to avoid contact by distorting the shape of their branches creating strangely shaped trees. This avoidance was visible clear in the samples where the eUBs were grafted in the MM without nicking of the epithelium of the CDs.

In general, wounding epithelia alters their production of signals.^17^ It may be that an altered signaling environment increases the probability of the two epithelia making contact, either through reduced production of repulsive factors or through production of attractive factors. It may also be that wound-derived signals promote some degree of epithelium-to-mesenchymal transition, or at least a weakening of existing cell-cell adhesions in nearby regions of the graft.

Whatever the mechanism of connection of the eUB with the host epithelial system, the fact that it can be induced to happen by a simple mechanical intervention raises an interesting new possibility for renal repair. In general, commentators discuss three strategies for repairing damaged kidneys; one involves promoting endogenous repair through, for example, mobilization of natural kidney stem cells.^18^ Another involves promoting integration of cells (typically, but not necessarily, stem cells) into existing, damaged kidney tissue to repair it,^19^and a third concentrates on generating entirely new kidneys from stem cells, for transplantation.^20^

The observation that grafted stem cell-derived epithelia can connect to host epithelia and integrate in the tissue as a part of it, raises the intriguing possibility of constructing new tissues to replace just parts of damaged kidneys and grafting them in to connect to the collecting duct/pelvis of the host organ. This might also can be useful in repairing other parts of the lower urinary tract such as urinary bladder and urethra, based on what has been published on the use of Wolffian duct-derived cells to repair bladder, by Joseph and colleagues, 2018.^21^ Of course, this would depend on connections and integrations being possible even in mature host kidneys – the experiments we have presented here all use very immature fetal material – but this is a question worth exploring in the future.

## Materials and Methods

### Induction of ureteric bud differentiation

A Hoxb7-GFP mouse ES line^11,22^ was obtained as a gift from Professor Ryuichi Nishinakamura’s laboratory, Kumamato University, Japan. Cells were maintained in mESC culture media which contains GMEM (Sigma G5154) supplemented with 10% FBS, GlutaMAX (1x, Gibco), MEM-NEAA (1x, Gibco), sodium pyruvate (1 mM, Gibco), β-mercaptoethanol (0.1 mM), and leukemia inhibitory factor (LIF, 1 U/μl, Santa Cruz sc-4989). The cell differentiation was performed using a previously published protocol.^11^ At day 10 of the differentiation, the EBs developed numerous ES cell-derived UB-like structures, which in this paper we refer to as engineered UBs (‘eUBs’).

### Grafting of eUBs into host kidneys using a modified grafting technique

At day 11.5, pregnant CD1 mice were sacrificed according to methods listed under Schedule One of the UK Animals (Scientific Procedures) Act, this was performed by trained UK Home Office license holders. E11.5 kidneys were isolated from CD1 mice embryos and the rudiments were cultured on 24 mm, 0.4 μm-pore membranes (Transwells, Corning 3450) in kidney culture medium (KCM) comprising Minimum Eagle’s Medium with Earle’s salts (MEM; Sigma M5650) with 10% fetal bovine serum (FBS) and 1% penicillin/streptomycin. The mESC-derived eUBs were isolated manually from day 10 spheroids using sharpened tungsten needles. Then, a single cut was introduced into either one branch of the ‘T’ within a host E11.5 cultured kidney, or into the shaft of the ureteric bud stalk of E11.5 kidneys, using a sharpened Tungsten needle. Then, an eUB (one end of which had also been cut, as a result of its separation from day 10 spheroids) was placed in contact with the site of the cut in the natural ureteric bud stalk or collecting duct. The grafted kidneys were incubated at 37°C, in 5% CO_2_ for 5 days for the MM grafts and 9 days for the PWM grafts in KCM, medium change every other day.

### Immunofluorescence stain

Samples were fixed with cold methanol for 30 mins at room temperature. They were washed with phosphate buffered saline (PBS) and blocked in staining buffer, containing 5% bovine serum albumin (BSA) in PBS, overnight at 4°C. For PKC-zeta antibody staining, samples were fixed in 4% PFA for 30 minutes in room temperature. Then, they were blocked using 5% BSA contains 0.2% Triton X-100 (sigma) overnight in 4°C. The same blocking buffer was used to prepare the primary antibody solution.

### Statistics

For categorical data (feature present/absent), 95% confidence intervals were calculated as ±1.96 [√ (p(1 − p)/n)] + 1/2 n.^23^

## Supplementary Material

Supplemental Material
