# Supplementary material for: Connection of ES Cell-derived Collecting Ducts and Ureter-like Structures to Host Kidneys in Culture
Source: Organogenesis. 2021 Sep 27;17(3-4):40–9. doi: 10.1080/15476278.2021.1936785 (PMC9208768; doi:10.1080/15476278.2021.1936785)
Supplement: Supplemental Material [file KOGG_A_1936785_SM7717.zip › supplementary/downloadFromZipFile.pdf]

Supplementary information for

Connection of ES cell-derived collecting ducts and ureter-like structures to host kidneys in culture

Supplementary figure1:

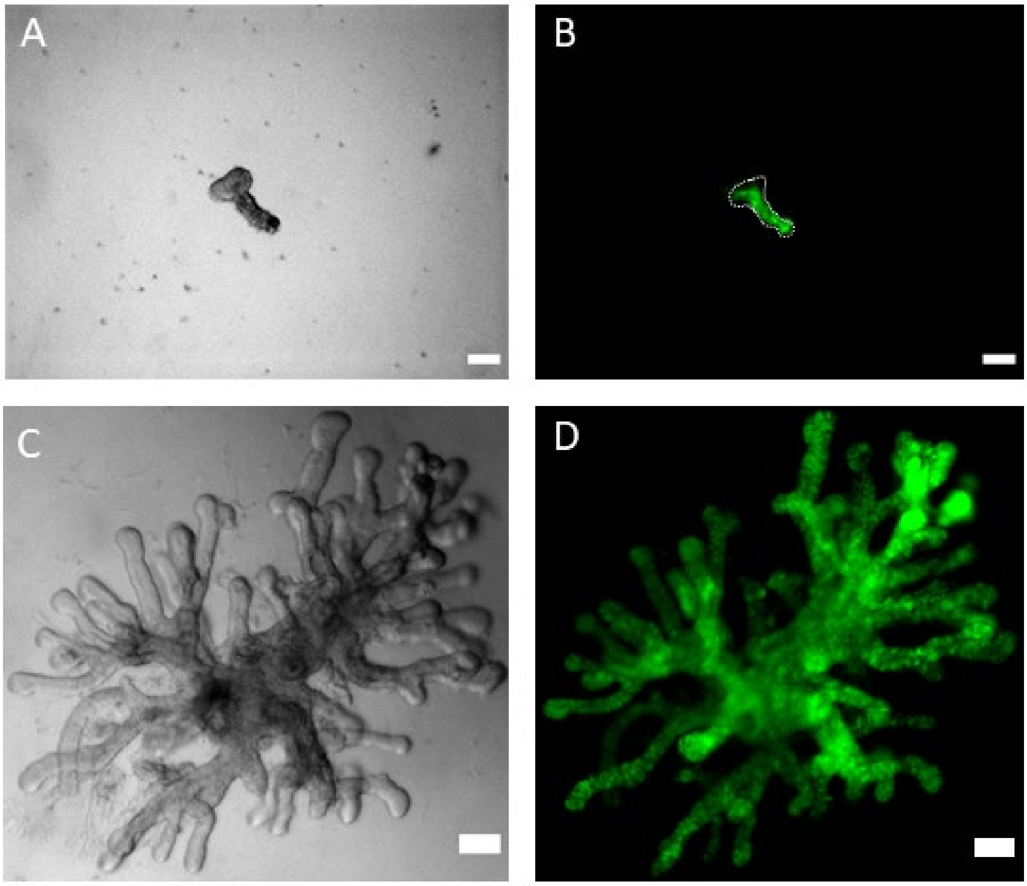

Figure 1S. Differentiation of ES cell derived eUBs using the Taguchi protocol. (A) Bright field image of an eUB dissected from day 10 Taguchi spheroids. **(B)** The GFP channel of A showing green HoxB7 expression in the dissected eUB. **(C)** Bright field image of eUB branching after 7 days in 3D gel supplemented with branching mixture. **(D)** The GFP channel of C, showing green HoxB7 expression in the branching eUB.

Supplementary table 1

Antibody list:

**Antibody**	**Host Species**	**Supplier & catalogue number**	**Working dilution**
Alpha-Smooth muscle actin	Goat	Novus nb300-978	1/100
Jagged 1	Goat	R&D AF599	1/100
Krt8	Rat	DSBH repository TROMA-1	1/100
Uroplakin	Rabbit	Gift from Tung-Tien Sun, New York	1/500
WT1	Rabbit	Abcam ab89901	1/100
Anti-PCK zeta	Rabbit	Abcam ab59364	1/100
Podocalyxin	Goat	AF1556 (R&D)	1/100
Anti-Rabbit 594	Donkey	Invitrogen 21207	1/100
Anti-Rabbit 488	Donkey	Invitrogen 21206	1/100
Anti-Rabbit 647	Donkey	Invitrogen A-31573	1/100
Anti-Goat 488	Donkey	Invitrogen A-11055	1/100
Anti-Goat 594	Donkey	Invitrogen A-11058	1/100
Anti-Goat 647	Donkey	Invitrogen A-21447	1/100
Anti-Rat 594	Chicken	Invitrogen A-21471	1/100
Anti-Rat 488	Donkey	Abcam Ab150153	1/100
